# Development of opioid analgesic tolerance in rat to extended-release buprenorphine formulated for laboratory subjects

**DOI:** 10.1371/journal.pone.0298819

**Published:** 2024-03-21

**Authors:** Christina M. Larson, Cecilia Barajas, Kelley F. Kitto, George L. Wilcox, Carolyn A. Fairbanks, Cristina D. Peterson

**Affiliations:** 1 Comparative and Molecular Biosciences, University of Minnesota College of Veterinary Medicine, St Paul, MN, United States of America; 2 Department of Experimental and Clinical Pharmacology, University of Minnesota College of Pharmacy, Minneapolis, MN, United States of America; 3 Department of Neuroscience, University of Minnesota Medical School, Minneapolis, MN, United States of America; 4 Department of Pharmacology, University of Minnesota Medical School, Minneapolis, MN, United States of America; 5 Department of Dermatology, University of Minnesota Medical School, Minneapolis, MN, United States of America; 6 Department of Pharmaceutics, University of Minnesota College of Pharmacy, Minneapolis, MN, United States of America; University of Texas Medical Branch at Galveston, UNITED STATES

## Abstract

Buprenorphine in an extended-release formulation intended for use in laboratory subjects is frequently administered to rats to provide extended analgesia without repeated handling. While levels of buprenorphine may persist in serum once extended-release buprenorphine has been introduced, exposure to opioids can cause opioid tolerance or opioid-induced hypersensitivity. This work examined the analgesic duration and efficacy of a single administration of extended-release buprenorphine intended for use in laboratory subjects in models of inflammatory pain and post-operative pain and the development of opioid tolerance in rat. After subcutaneous administration of 1 mg/kg extended-release buprenorphine, analgesic efficacy did not persist for the expected 72 hours. No changes were observed in mechanical thresholds in the hindpaws that were contralateral to the injury, suggesting a lack of centrally mediated opioid-induced hypersensitivity. To determine whether opioid tolerance arose acutely after one exposure to extended-release buprenorphine, we conducted the warm water tail flick assay; on Day 1 we administered either saline or extended-release buprenorphine (1 mg/kg) and on Day 3 we quantified the standard buprenorphine dose-response curve (0.1–3 mg/kg). Rats previously given extended-release buprenorphine displayed decreased analgesic responses after administration of standard buprenorphine as compared to the robust efficacy of standard buprenorphine in control subjects. Males appeared to show evidence of acute opioid tolerance, while females previously exposed to opioid did not demonstrate a decreased response at the doses examined. Taken together, these results suggest that opioid tolerance arises quickly in male rats after exposure to the extended-release formulation of buprenorphine. This tolerance may account for the brief period of antinociception observed.

## 1. Introduction

Buprenorphine hydrochloride is the most common analgesic used to provide post-operative pain relief in laboratory rats [1]. It is an opioid that provides rats with an intermediate level of analgesia when compared to morphine and butorphanol [2] and its efficacy has been demonstrated in post-operative pain [3] as well as in inflammatory and neuropathic pain states [4].

Analgesic options for the laboratory rat include nonsteroidal anti-inflammatories (NSAIDs), local anesthetics, and opioids, but only a limited subset are viable for analgesia in rats. Local anesthetics such as lidocaine require precise injection and may be difficult to administer optimally even in species larger than a rat. Topical formulations may be diverted from the intended site of action and ingested via grooming. NSAIDs, including ketoprofen, may give rise to perforating gastric ulcers and can therefore be lethal [5]. Opioids can induce not only analgesic tolerance but respiratory depression and death [6]. However, the ceiling effect of buprenorphine appears to resolve these risks [7–9].

Handling rats to provide repeated injections for post-operative analgesic care can alter research results [10,11]; thus, analgesics with longer duration of action are preferred. Extended-release formulations are advantageous, particularly for addressing pain arising from surgery [11]. Extended-release formulations of buprenorphine have been introduced; initial work evaluated analgesia in male rats using the tail flick assay in naïve animals and in subjects with a surgically created unicortical tibial defect [12], in female rats after laparotomy [13], and for safety [9,14]. To our knowledge, rats given buprenorphine in extended-release formulations have not been assessed for side effects including opioid tolerance (where escalated opioid administration is needed to achieve previously observed analgesia) or opioid hypersensitivity (an increase in pain or sensitivity following opioid administration [15]).

This study was designed to determine the duration of analgesia induced by extended-release buprenorphine using various pain models in rats. It is an extension of work previously performed in the mouse [16] to determine whether the acute opioid tolerance we observed after single administration of extended-release buprenorphine occurs in other species. We chose (1) the complete Freund’s adjuvant model for inducing inflammation and (2) an incisional model akin to minor surgery to recapitulate modest post-operative pain as the most clinically relevant models, with an expected time course of analgesia up to 72 hours. We detected limited duration of analgesia that appeared to differ between sexes. To quantify opioid tolerance, we examined sensory responses in naïve animals first administered extended-release buprenorphine or saline followed by standard buprenorphine. We observed a depressed dose-response curve in males, but not in females. Extended-release buprenorphine produced limited analgesic duration, no contralateral hypersensitivity, and a decreased dose-response curve after initial exposure. These findings indicate that acute opioid tolerance occurs in rats after one injection of extended-release buprenorphine formulated for use in laboratory subjects.

## 2. Materials and methods

These studies were conducted following the National Institutes of Health *Guide for the Care and Use of Laboratory Animal Species*, the United States Public Health Service Policy, and the Animal Welfare Act. These procedures were reviewed and approved by the Institutional Animal Care and Use Committee (IACUC) at the University of Minnesota on protocols 1703-34617A and 1908-37350A. The University of Minnesota receives accreditation from the Association for the Assessment and Accreditation of Laboratory Animal Care (AAALAC).

### 2.1 Animal subjects

Specific pathogen free Hsd:Sprague-Dawley rats weighing 175-260g were purchased from a commercial vendor (Envigo, USA), and were housed as male-male or female-female pairs. The environment was humidity- and temperature-controlled under a light/dark cycle at a ratio of 14:10. All subjects had ad lib access to water and food. Subjects were included in one experiment only.

### 2.2 Chemical agents

We purchased complete Freund’s adjuvant (CFA) from Sigma Chemical (Sigma-Aldrich Company, Saint Louis, Missouri, USA), and extended-release buprenorphine (formerly named sustained-release buprenorphine) [1 mg/mL] from ZooPharm (Windsor, Colorado, USA), which was subsequently acquired by Wedgewood Pharmacy (Swedesboro, New Jersey, USA). There has been no alteration in formulation associated with the renaming of sustained-release buprenorphine to extended-release buprenorphine. We obtained buprenorphine hydrochloride (Par Pharmaceutical, Chestnut Ridge, New York, USA) and isoflurane (Piramel Enterprises Limited, Andhara Pradesh, India) from Midwest Veterinary Supply (Lakeville, Minnesota, USA). We used sodium chloride (also purchased from Sigma Chemical) to make 0.9% saline which was sterile-filtered through a 0.22-micron cellulose acetate filter (Corning Incorporated, Corning, New York, USA).

### 2.3 Experimental design

Experimenters were blinded to the substance given and dose used. Baseline thresholds were measured for each subject prior to introduction of any experimental variable. All injections were subcutaneous injections. All treatment and control groups received an equivalent injection volume; control animals were administered an equal volume of saline on a mL/kg basis according to the quantity of extended-release buprenorphine they would have received if they had been in the treatment group. To minimize the opportunity for retrogradation of the administered substance along the needle tract after needle withdrawal, the needle was briefly held in place for 30–60 seconds following substance administration and gentle pressure was applied at the injection site. An electronic von Frey anesthesiometer (Bioseb In Vivo Research Instruments, North Pinellas Park, Florida, USA) was used to measure the mechanical threshold of tactile hypersensitivity in the inflammatory and post-operative models. As a within-subject control, the mechanical threshold for the contralateral paw was also measured. After establishment of tactile hypersensitivity, each subject received extended-release buprenorphine or saline and sensory assays were performed at defined time points. Negative controls were either a saline group, the contralateral paw, or both, as indicated in each model. To assess acute thermal nociception in subjects, tail flick latency was recorded. Timelines are provided for the tail flick assays, outlining dose, treatment, and time of administration.

### 2.4 Complete Freund’s adjuvant-induced inflammatory hypersensitivity

Inflammatory pain was modeled by administering complete Freund’s adjuvant (CFA) [17] via intraplantar injection to induce inflammation in the hindpaw. First, rats were evaluated for mechanical sensitivity on ipsilateral and contralateral hindpaws prior to introduction of CFA. Then, isoflurane at 2.5% was administered to effect. Once a plane of surgical anesthesia had been achieved, one hindpaw (the ipsilateral paw) received a single intraplantar injection comprised of 50 μL of complete Freund’s adjuvant (CFA) diluted 1:1 with saline. Once isoflurane anesthesia ceased, each subject was recovered from anesthesia in the span of 5–10 minutes and mobile. Complete Freund’s adjuvant induces an inflammatory hypersensitivity which is fully developed within 3 days. Mechanical thresholds were again assessed to confirm the development of tactile hypersensitivity, then each subject in a treatment group subsequently received 1 mg/kg subcutaneous (SC) extended-release buprenorphine, while control subjects received the equivalent volume of subcutaneous saline. Contralateral paws were also assessed in all groups. Mechanical hypersensitivity on ipsilateral paws was assessed at 1, 2, 3, and 24 hours after injection in the first cohort, and at 2, 4, 8, and 24 hours in the second cohort.

### 2.5 Incisional model of post-operative tactile hypersensitivity

As previously described by Brennan and colleagues, the post-operative model induces tactile hypersensitivity rapidly following a plantar surgical incision [18–20]. First, rats were evaluated for baseline mechanical thresholds. Isoflurane was used to effect to anesthetize each animal for surgery. A 3–5 mm longitudinal incision was made through the skin and into the fascia of the underlying plantar muscle. For closure of the incision, undyed braided coated polyglycolic acid suture in 3–0 size (Polysun™, Angiotech, USA) was used. After isoflurane anesthesia was discontinued, animals recovered within 5–10 minutes. To recapitulate common clinical veterinary recommendations for analgesia to be provided during or immediately following a surgery, extended-release buprenorphine (1 mg/kg) or a negative control consisting of an equivalent volume of saline was administered SC immediately upon anesthetic recovery rather than taking time to quantify developing hypersensitivity pursuant to the new incision. Subsequently, each subject was assessed for mechanical hypersensitivity at time points 1, 2, 3, 24-, and 48-hours following incision. As within-subject controls, contralateral paws were assayed congruently.

### 2.6 Tactile assessment of mechanical thresholds

To quantify the development and persistence of tactile hypersensitivity, the mechanical thresholds of the rats were measured. The testing apparatus utilized plastic enclosures on an elevated mesh screen, where rats were acclimated for 15–30 minutes prior to sensory assessment. The anesthesiometer probe was gently applied to each hindpaw. To terminate the application of pressure, the subject briskly withdrew the paw (in seconds), and the pressure threshold which prompted withdrawal was recorded. Sensory assessments took place at specified time points after administration of experimental compound or control as listed for each model.

### 2.7 Acute thermal nociception assessed by tail flick latency in warm water bath

To assess thermal nociception, tails were immersed in a water bath of 52.5°C, and the latency for the subject to flick or remove their tail was recorded in seconds. Tails were removed from the bath at a pre-determined cut-off time of 12 seconds if not already voluntarily withdrawn [21]. First, rats were assessed for baseline sensitivity. The treatment group received SC extended-release buprenorphine (1 mg/kg); the control group received an equal volume of SC saline. This assay was subsequently performed to assess post-administration sensitivity at the following timepoints: 10 minutes, 15 minutes, 30 minutes, 1 hour, 2 hours, 3 hours, and 24 hours after compound administration.

### 2.8 Standard buprenorphine dosing to test for buprenorphine dose-response

To assess the efficacy of standard buprenorphine in the rats previously given extended-release buprenorphine, the latency of each subject to tail flick was assayed as described above. First, rats were assessed for baseline sensitivity. On Day 1, a subject received either 1 mg/kg SC extended-release buprenorphine or an equivalent volume of saline (for the negative control group). On Day 3, both the extended-release buprenorphine group and the negative control group were then administered increasing doses (0.1 mg/kg, 0.3 mg/kg, 1 mg/kg, and 3 mg/kg) of standard buprenorphine, and tail flick latency was assessed 30 minutes after each dose. First, the lowest dose (0.1 mg/kg) was administered, and tail flick latency was assessed. Then the following dose (0.3 mg/kg) was administered, and subjects assessed again. Each additional dose administered increased in a stepwise fashion.

### 2.9 Activity monitoring

Rat general activity was tracked and measured on three separate occasions—before CFA or incision, 24-hours after receiving CFA, and again 24-hours after receiving either saline or 1 mg/kg s.c. extended-release buprenorphine. Rats were allowed at least 30 minutes to acclimate to the behavior room before beginning testing. During 10-minute sessions, rats were placed into acrylic activity boxes (27.50” x 8.75” x 8.00”, made in house) and their activity was recorded using Logitech StreamCams. Distance traveled, mean speed, max speed, time immobile, and immobile episodes were automatically detected using AnyMaze video tracking software version 7.32.

### 2.10 Statistical analysis

Experimental groups consisted of a minimum of 12 animals (6 females and 6 males). No subjects were excluded from either the experiments or the analysis. To illustrate mechanical thresholds, figures present the data as mean +/- standard error of the mean (SEM) in grams of pressure. In tail flick assays, the standard method of illustrating the data is to present it as percent maximum possible efficacy (%MPE), which these figures also follow. This method permits calculating dose-response curves as well as comparing relative potency between assessments and between cohorts. MPE is calculated thusly: [(tail flick latency at a given time point—baseline latency)/(cut-off time—baseline latency)], summarized as %MPE = (TL-BL) / (CO–BL), where TL indicates seconds of tail flick latency at a given time point, BL is the baseline latency (seconds), and CO is the cut-off time (12 seconds).

The significance for any individual pair-wise comparison was set at P < 0.05. Every multiple-comparison analysis included post hoc correction set as α = 0.05. In the initial inflammatory cohort consisting only of a treatment group, a 1-way repeated measures analysis of variance (ANOVA) was conducted to compare the initial inflammatory hypersensitivity to subsequent time points after administration of extended-release buprenorphine, with Bonferroni’s post hoc correction to adjust for multiple comparisons. The hypersensitivity between extended-release buprenorphine and saline groups in the inflammatory model was analyzed with 2-factor (time and treatment) repeated measures ANOVA with Bonferroni post hoc correction. The post-operative model was also analyzed with the same statistical tests. All ANOVAs included the Geisser-Greenhouse correction to accommodate potential unequal variances between groups. To analyze tail flick data, Welch’s unequal variance unpaired t-tests with Bonferroni post hoc correction was used for pair-wise comparisons between groups at each time point. To analyze activity monitoring data, a repeated measures one-way ANOVA with the Geisser-Greenhouse correction and Bonferroni multiple comparisons test was performed to compare each assessment. The software GraphPad Prism 9.1.0 (GraphPad Software, San Diego, California, USA) was used to perform data analysis and graphical representation.

## 3. Results

### 3.1 Tactile hypersensitivity

#### 3.1.1 Complete Freund’s adjuvant model of inflammatory tactile hypersensitivity

We used the CFA model to assess the analgesic efficacy of extended-release buprenorphine against inflammatory algesia. Left hindpaws received CFA (ipsilateral hindpaw), while right hindpaws (contralateral hindpaw) did not. Responses are presented as grams of pressure. Contralateral thresholds were not utilized within statistical analysis but are reported below.

**Fig 1:**
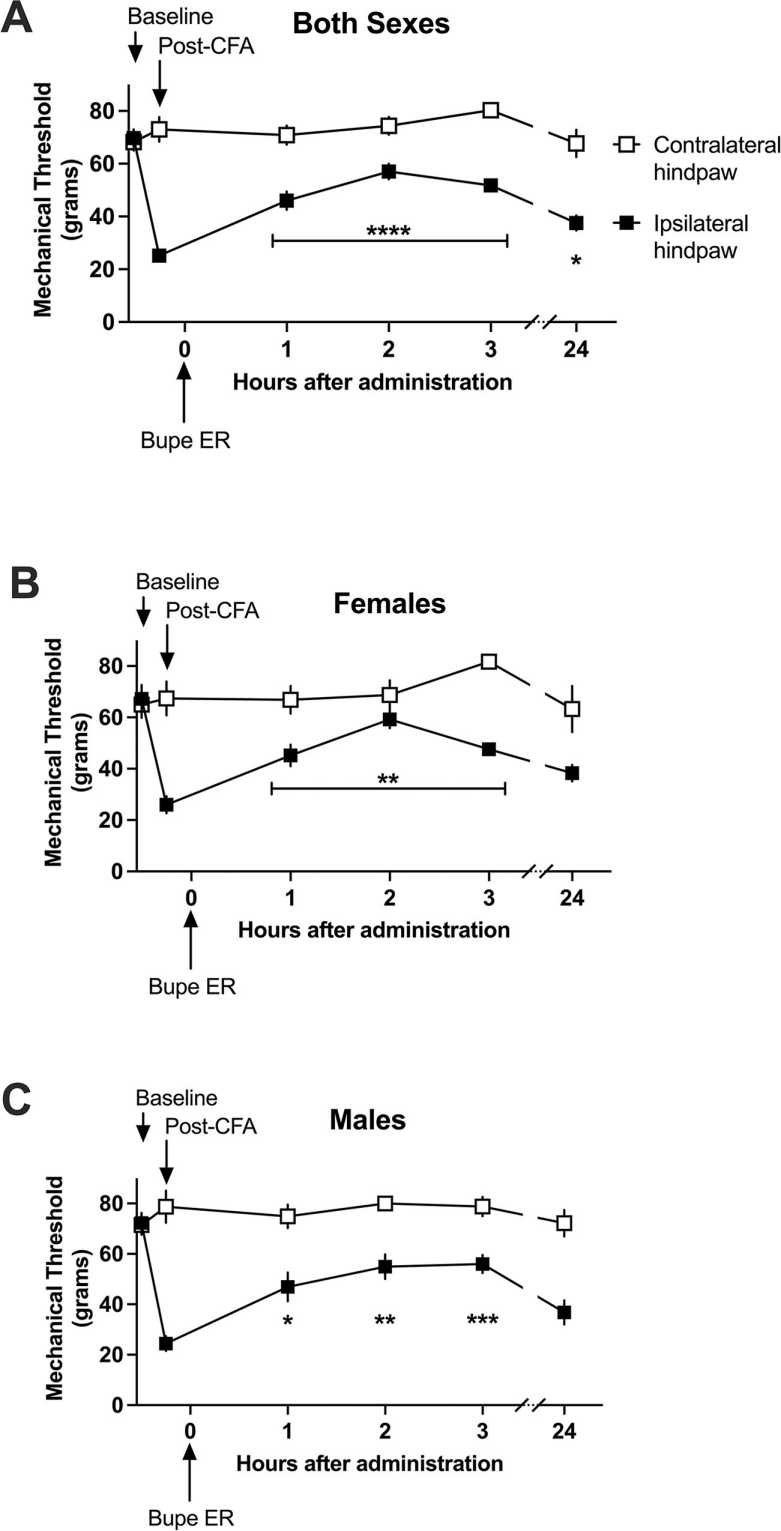
Effect of extended-release buprenorphine (1 mg/kg SC) on tactile hypersensitivity in a rat model of inflammation. The mechanical nociceptive threshold is transiently changed by extended-release buprenorphine. Initial hypersensitivity tested at time point 0 was compared to time points following administration of extended-release buprenorphine (Bupe ER). Responses are measured in grams of pressure to quantify the mechanical threshold. Data presented as means of 16 rats (8 males, 8 females) +/-S.E.M and compared with one-way repeated measures ANOVA with Bonferroni post hoc correction. *P < 0.05, **P < 0.01, ***P < 0.001. A) Bupe ER increased mechanical thresholds compared to the initial, CFA-induced initial hypersensitivity at 1, 2, 3, and 24 hours after administration. B) In females analyzed alone, mechanical thresholds were significantly elevated 1, 2, and 3 hours following Bupe ER injection. However, there was no significant difference from post-CFA baseline at 24 hours post-injection of Bupe ER. C) In males analyzed alone, mechanical thresholds were significantly elevated 1, 2, and 3 hours following Bupe ER injection. However, there was no significant difference from post-CFA baseline at 24 hours post-injection of Bupe ER.

First, we assessed the change in tactile paw withdrawal thresholds relative to the initial hypersensitivity induced by complete Freund’s adjuvant (Fig 1).

Using the one-way repeated measures ANOVA with Bonferroni post hoc correction, we analyzed the effect of extended-release buprenorphine on tactile hypersensitivity. Initial hypersensitivity was compared to hypersensitivity after injection of extended-release buprenorphine. In the cohort of males and females combined (Fig 1A), there was a significant effect of extended-release buprenorphine, with a significant difference between the initial hypersensitivity and the hypersensitivity 1, 2, and 3 hours after administration (P < 0001). This effect remains statistically significant at 24 hours post-administration (P < 0.05), though is of a low magnitude of effect. These data appear to support that extended-release buprenorphine (1 mg/kg SC) imparts a transient analgesic reduction of hypersensitivity, though of reduced magnitude of effect as early as 24 hours following administration of extended-release buprenorphine.

In female subjects analyzed alone (Fig 1B), extended-release buprenorphine significantly elevated the mechanical thresholds for 1, 2, and 3 hours following administration (P < 0.01); however there is no observed efficacy at 24 hours post-administration. In male subjects analyzed alone (Fig 1C), there was again significantly elevated mechanical thresholds for 1, 2, and 3 hours following administration (P < 0.05, 0.01, and 0.001 respectively). However, thresholds again return to be not significantly different from baseline thresholds by 24 hours following administration of extended-release buprenorphine. These sex-segregated data suggest that while there is initial efficacy of extended-release buprenorphine at alleviating inflammatory hypersensitivity, this efficacy is reduced or abolished by 24 hours following administration.

**Fig 2:**
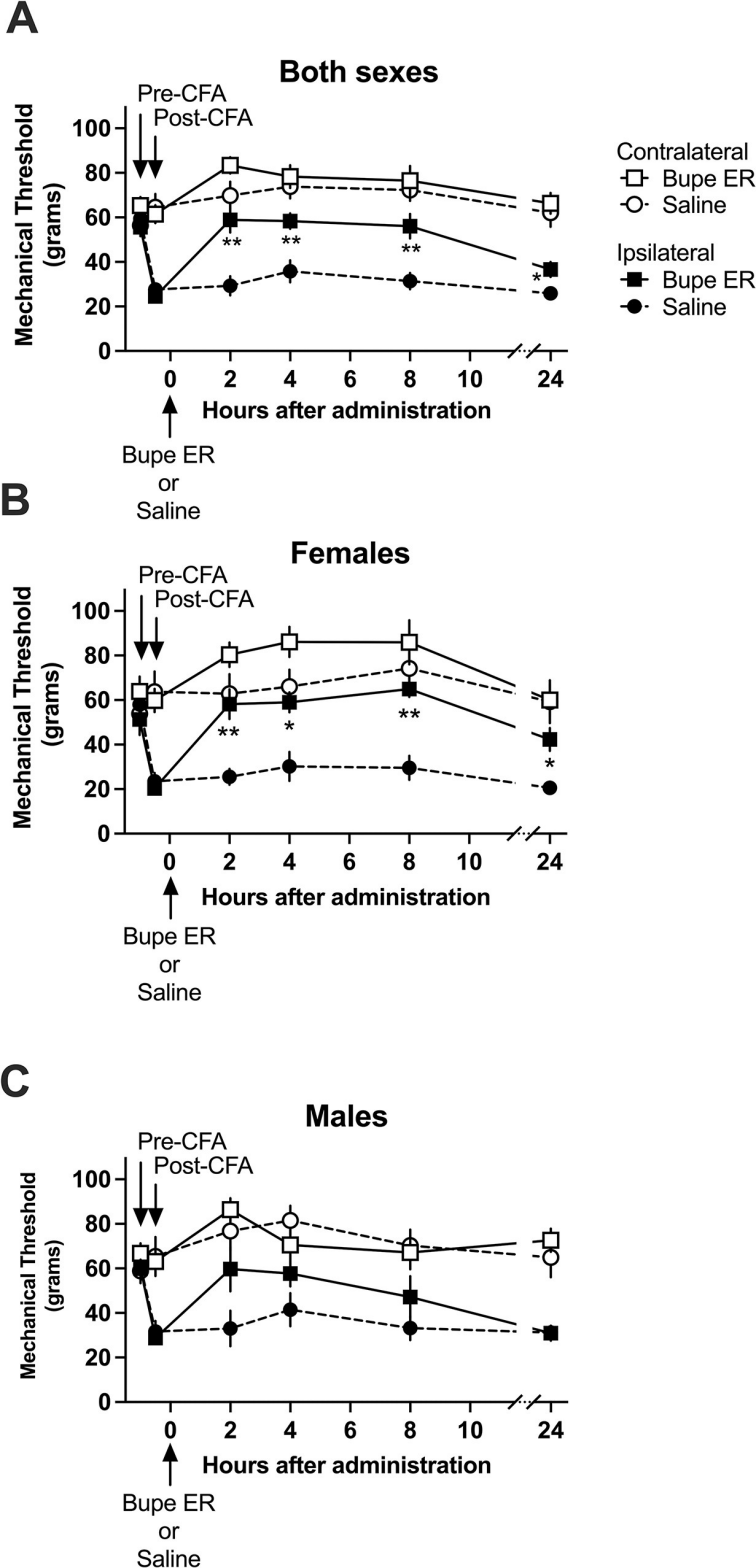
Effect of extended-release buprenorphine (1 mg/kg SC) as compared to saline control on tactile hypersensitivity in a model of inflammatory hypersensitivity in rats. Mechanical thresholds are shown as grams of pressure. Data are presented as means of 12 rats (6 males, 6 females) +/-S.E.M and compared with two-way repeated measures ANOVA with Bonferroni post hoc correction. *P < 0.05, **P < 0.01. A) In both male and female subjects analyzed together, there is significant elevation of mechanical thresholds for rats treated with extended-release buprenorphine (Bupe ER) as compared to rats treated with saline following induction of inflammation via intraplantar CFA. B) In female subjects analyzed alone, rats treated with Bupe ER show significantly increased mechanical thresholds as compared to saline-treated rats. C) In male subjects analyzed alone, there is no statistically significant difference between the treatment groups at any time point.

Next, we assessed the change in hypersensitivity relative to a saline control group in the same inflammatory hypersensitivity state induced by complete Freund’s adjuvant (Fig 2). We additionally tested at points between 3 and 24 hours to assess the time course of efficacy of the extended-release buprenorphine administered at 1 mg/kg SC.

To determine whether extended-release buprenorphine causes a measurable alleviation in tactile hypersensitivity in the treatment group compared to the saline control group at any time point, we used the two-way repeated measures ANOVA with the post hoc correction of Bonferroni’s multiple comparisons method. In both male and female subjects analyzed together (Fig 2A), we again saw efficacy of extended-release buprenorphine, this time assessed at 2, 4, 8 (P < 0.01), and 24 hours (P < 0.05) following administration. While the mechanical thresholds of extended-release buprenorphine-treated subjects are significantly elevated as compared to saline-treated control subjects; the magnitude of alleviation of hypersensitivity is again quite small by 24 hours post-administration of extended-release buprenorphine.

In female subjects analyzed alone (Fig 2B), extended-release buprenorphine-treated subjects again displayed significantly elevated mechanical thresholds as compared to saline-treated subjects (P < 0.01 at 2 and 8 hours, P < 0.05 at 4 and 24 hours). However, in male subjects analyzed alone (Fig 2C), there was no significant overall effect of extended-release buprenorphine distinct from saline at any assessment. These data suggest that 1 mg/kg SC extended-release buprenorphine does not yield an analgesic effect significantly different from saline in males at any measured time.

Taken together, the studies presented in Figs 1 and 2 support that extended-release buprenorphine imparts only a moderate reduction of inflammation-induced tactile hypersensitivity in the rat with a notable lack of efficacy in male subjects. This moderate reduction may be attributable to extended-release buprenorphine-induced opioid tolerance as further described in 3.2.1 and 3.2.2.

#### 3.1.2 Activity monitoring of inflamed subjects

**Fig 3:**
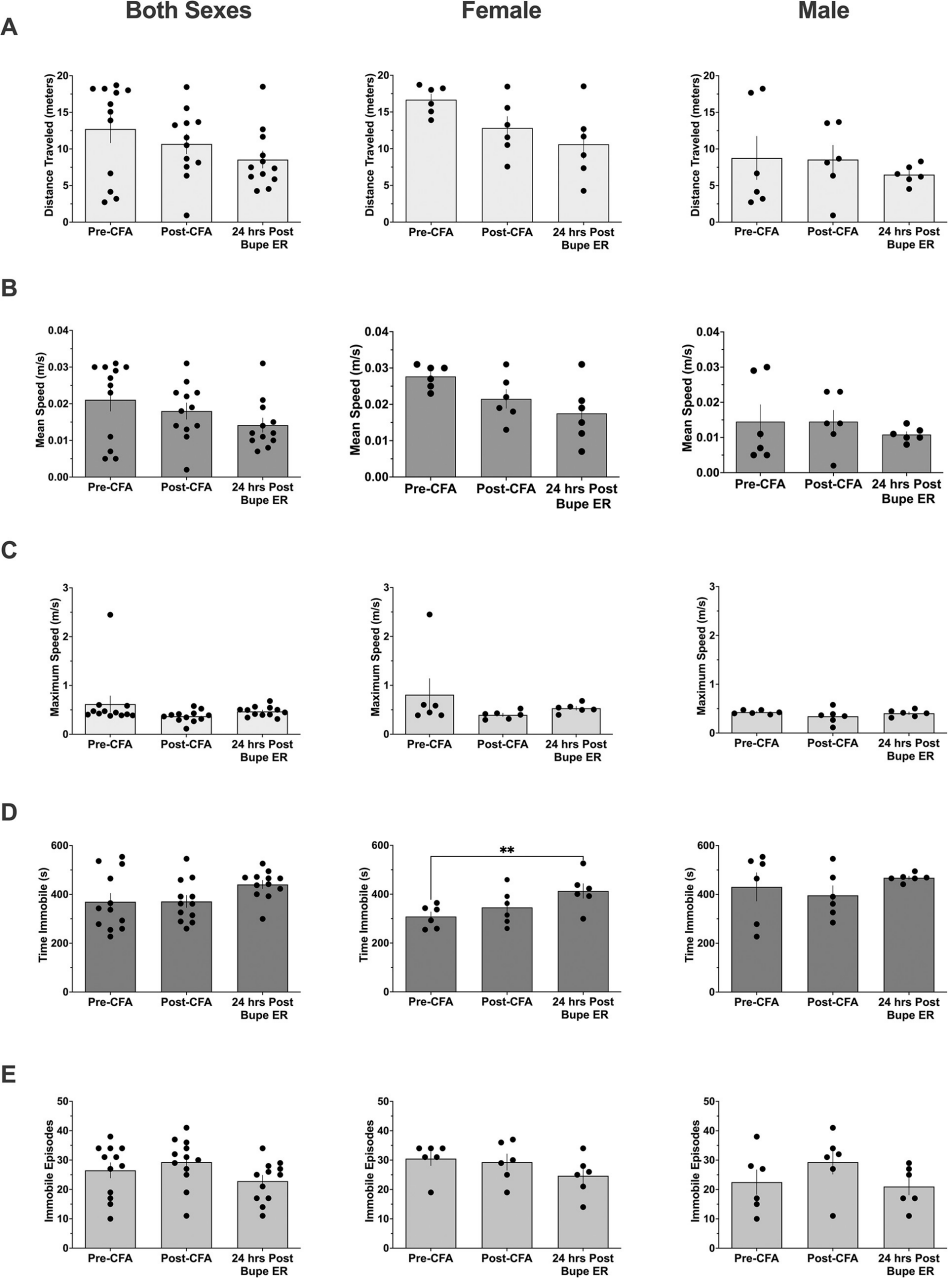
Activity monitoring of CFA-inflamed and Bupe-ER treated rats. Each rat (6 males and 6 females) underwent 3 recording sessions: prior to intraplantar CFA injection (Pre-CFA), 24 hours following intraplantar injection of CFA (Post-CFA), and 24 hours after injection of 1 mg/kg Bupe ER s.c. (24 hrs Post Bupe ER). Each session yielded the following activity parameters for assessment: Distance Traveled (Meters, A), Mean Speed (m/s, B), Maximum Speed (m/s, C), Time Immobile (seconds, D), and number of Immobile Episodes (E). The data were analyzed as a combination of both sexes (leftmost colum of graphs), and then separated into female only (center column of graphs) and male only (right column of graphs). All data sets were analyzed by repeated measures one-way ANOVA with the Geisser-Greenhouse correction and Bonferroni post hoc correction, **p < 0.01.

Many efforts have been made to assess non-reflexive indications of pain and discomfort in laboratory subjects. To this end, we assessed the motor activity of rats prior to (Pre-CFA) and 24 hours following (Post-CFA) induction of local inflammation via intraplantar CFA injection. Finally, these same subjects were injected with extended-release buprenorphine at 1 mg/kg following their post-CFA assessment and were assessed for a third time at 24 hours following extended-release buprenorphine injection (24 hrs Post Bupe ER, Fig 3). All subjects were assessed for their total distanced traveled (meters, 3A), mean speed (meters/second, 3B), maximum speed (meters/second, 3C), time immobile (seconds, 3D), and the total number of immobile episodes (3E). A combined analysis examining both male and female subjects together (leftmost column of graphs) found no significant difference for any parameter 24 hours following CFA as compared to the baseline, Pre-CFA measurements. Additionally, there was no difference 24 hours following extended-release buprenorphine injection as compared to the Post-CFA measurements. Finally, there was no difference between Pre-CFA baselines as compared to 24 hours Post-CFA injection. When the analysis was split by the sex of the subject, only the time spent immobile was increased 24 hours following treatment with extended-release buprenorphine in the inflamed female subjects (middle column of graphs) as compared to their Pre-CFA baseline measurement (P < 0.01). While this reached statistical significance, the overall relevance is difficult to determine as the subjects did not travel a significantly lower total distance, did not travel at a lower speed, and did not have increased contralateral von Frey thresholds (Figs 1 and 2), suggesting that there was no detected sedative effect of the extended-release buprenorphine. In male subjects analyzed alone (rightmost column of graphs), there were no significant differences at any point of any measured parameter. Activity monitoring data were analyzed by repeated measures one-way ANOVA with the Geisser-Greenhouse correction and Bonferroni multiple comparisons correction.

#### 3.1.3 Incisional tactile hypersensitivity model

To evaluate extended-release buprenorphine in a clinically relevant model of post-operative pain, we utilized an incisional model. Mechanical paw withdrawal baseline assessments were conducted on both hindpaws, followed by surgical incision of the left hindpaw (ipsilateral hindpaw); the right hindpaws (contralateral to the incision) remained intact. Following surgery, left hindpaws displayed reduced paw withdrawal thresholds, i.e., mechanical hypersensitivity. The withdrawal thresholds of contralateral hindpaws were not altered following incision or drug administration, indicative of a lack of centrally mediated hypersensitivity.

**Fig 4:**
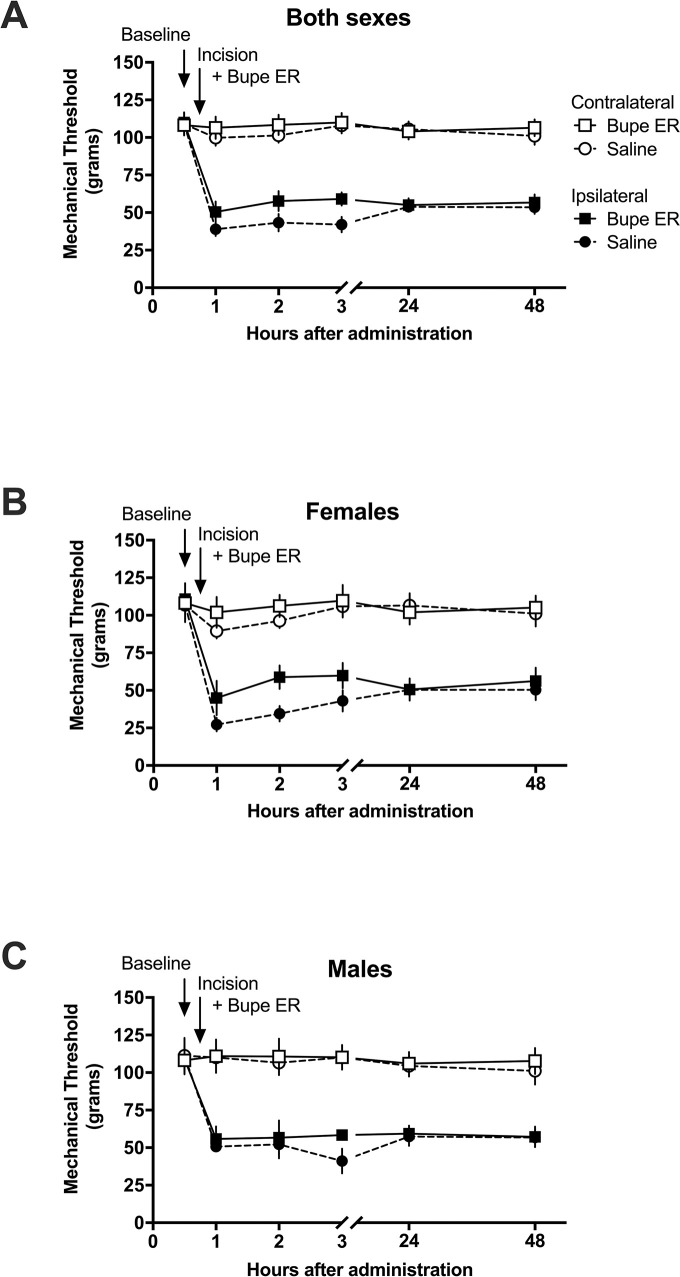
Effect of extended-release buprenorphine (1 mg/kg, SC) as compared to saline control on tactile hypersensitivity in an incisional model of post-operative pain in the rat. The mechanical nociceptive thresholds in subjects given extended-release buprenorphine are not significantly different from those in subjects given saline. Mechanical thresholds are shown as grams of pressure. Data are presented as means of 12 rats (6 males, 6 females) +/-S.E.M. Saline and extended-release buprenorphine groups were compared with two-way repeated measures ANOVA with Bonferroni post hoc correction. *P < 0.05. A) For male and female subjects analyzed togethere, there was no significant difference between the treatment groups. B) In females alone, there was no significant difference between the treatment groups. C) In males alone, there was no significant difference between the treatment groups.

To determine whether the alleviation of hypersensitivity differs from the untreated state, we used two-way repeated measures ANOVA (time–treatment), followed by Bonferroni’s multiple comparisons post hoc correction. The effect of extended-release buprenorphine on post-incision tactile hypersensitivity was compared between the saline group and the treatment group at each time point. In rats that received an intraplantar incision to model post-operative pain (Fig 4A), there was no significant difference between the ipsilateral paw of the two treatment groups at any time (all P > 0.05). When the data were separated by sex, there remained no significant differences between subjects that received saline and subjects that received extended-release buprenorphine at any timepoint for both the female subjects analyzed alone (Fig 4B) as well as the male subjects analyzed alone (Fig 4C). These data demonstrate that extended-release buprenorphine did not provide an analgesic effect that elevated mechanical thresholds to any degree distinct from saline in the post-incisional pain state.

### 3.2 Extended-release buprenorphine induces acute opioid tolerance

**Fig 5:**
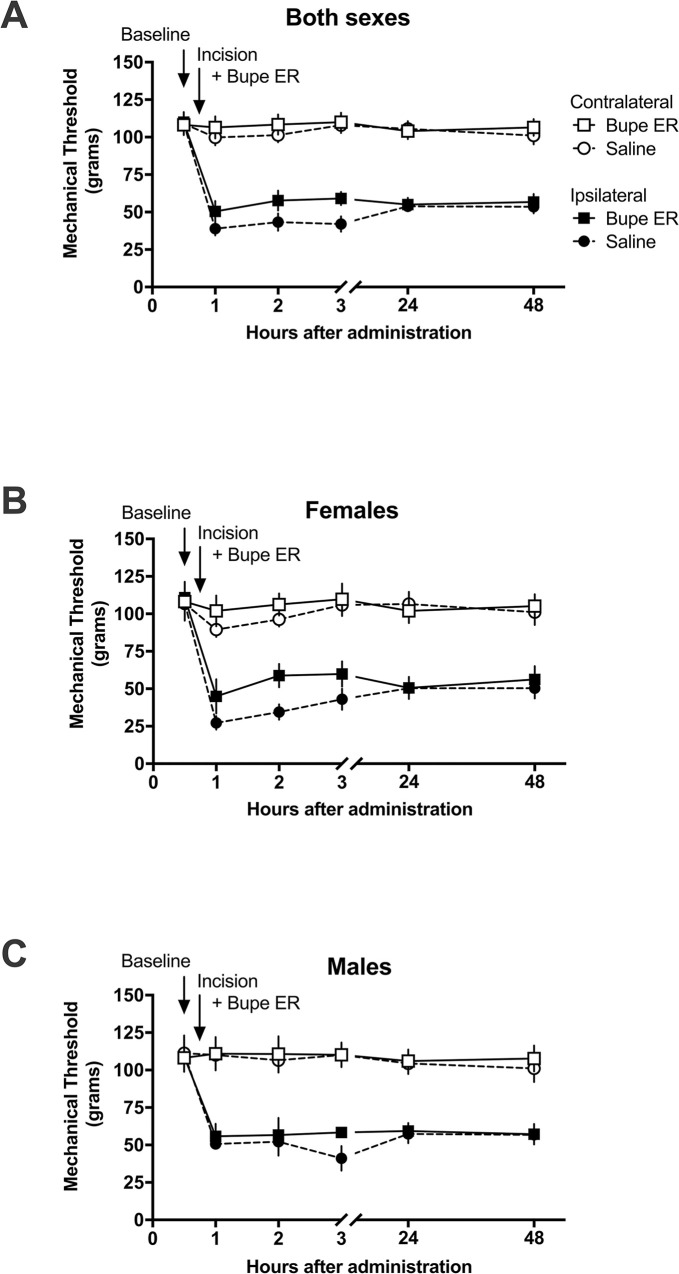
Study timeline for the warm-water tail flick assays. Day 1: Effects of extended-release buprenorphine on acute thermal nociception compared to saline. Day 3: Effects of increasing doses of standard buprenorphine (dose-response curve) comparing previous exposure to extended-release buprenorphine or previous exposure to saline.

To assess development of opioid tolerance, we examined tail flick latency in naïve subjects after immersion of tail tips in 52.5°C water. This assay reflects the threshold for acute thermal nociception and takes place in two stages (Fig 5). Day 1 assesses response to extended-release buprenorphine or saline, while Day 3 assesses response to standard buprenorphine given in escalating doses in both groups (prior opioid exposure vs. no prior opioid exposure). Subjects given extended-release buprenorphine (Day 1) were compared to subjects given saline (Day 1), and the comparison between groups at each time point was analyzed by Welch’s multiple unpaired t-test followed by the Bonferroni post hoc correction.

#### 3.2.1. Quantifying the antinociceptive effect of extended-release buprenorphine

**Fig 6:**
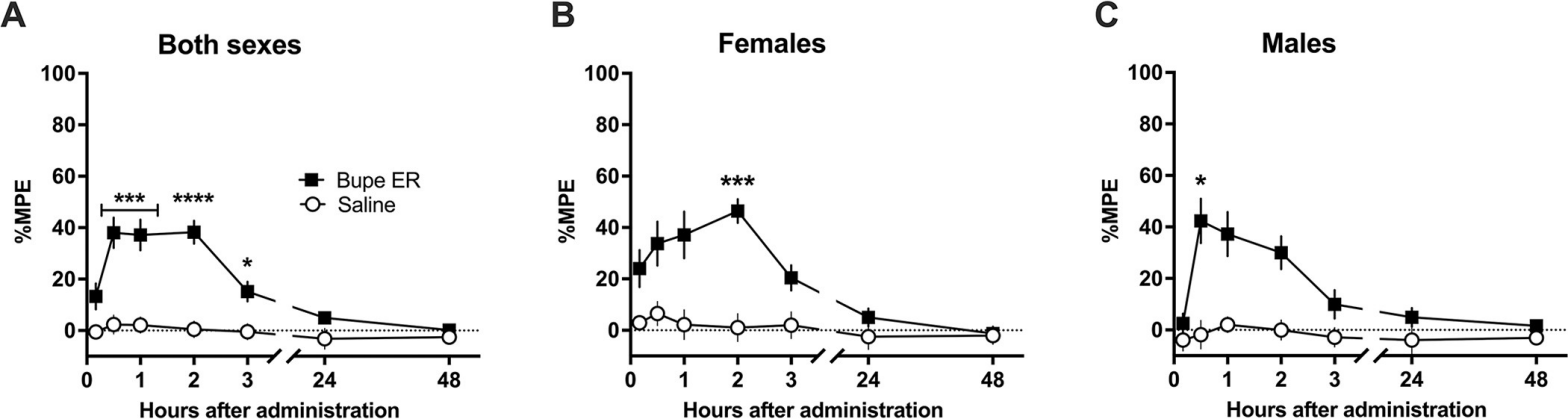
Effect of extended-release buprenorphine (1 mg/kg, SC) vs. saline on the acute thermal nociception in naïve rats, quantified by tail flick assay in 52.5°C warm water bath. Data presented as means of 12 rats (6 males, 6 females) +/-S.E.M and analyzed by Welch’s multiple unpaired t-tests followed by Bonferroni post hoc correction. *P < 0.05, ***P < 0.001, ****P < 0.0001. A) There is a significant difference between the effects of extended-release buprenorphine (Bupe ER) vs. saline demonstrated at 30 min, 1, 2, and 3 hrs after administration. There was no significant difference at 24 or 48 hours following administration. B) In female subjects analyzed alone, there is a significant difference at only 2 hrs following administration of Bupe ER as compared to saline. C) In male subjects analyzed alone, there is a significant difference at only 2 hrs following administration of Bupe ER as compared to saline.

First, we determined if extended-release buprenorphine produced an antinociceptive effect on the acute thermal nociception induced by the warm water bath (Fig 6).

Extended-release buprenorphine compared to saline significantly extended latency to tail flick in rats (Fig 6A) at 30 minutes, 1 hour (both P < 0.001), 2 hours (P < 0.0001), and 3 hours (P < 0.05) following administration of 1 mg/kg s.c. extended-release buprenorphine. No other time point was significantly different between groups.

In female subjects analyzed alone (Fig 6B), latency was significantly extended by 1 mg/kg extended-release buprenorphine at 2 hours (P < 0.001). No other time point was significantly different between groups. In male subjects analyzed alone (Fig 6C), tail flick latency was extended by extended-release buprenorphine only at 30 minutes after administration (P < 0.05). No other time point was significantly different between groups. These data indicate extended-release buprenorphine does have an antinociceptive effect on acute thermal nociception, though this effect resolves by 24 hours following administration.

#### 3.2.2 Analysis of dose-response curve

**Fig 7:**
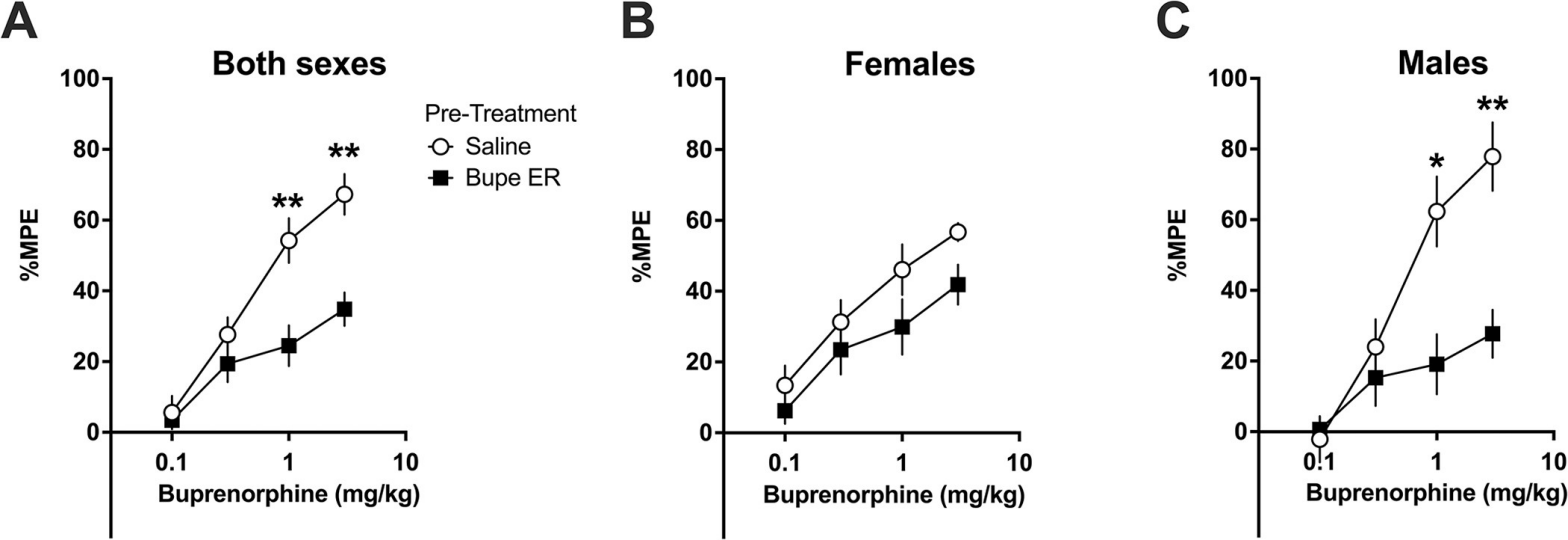
Effect of previous administration of extended-release buprenorphine (1 mg/kg SC) or saline in rats using a warm-water tail flick assay to assess acute thermal nociception. Standard buprenorphine administered consecutively with absolute dosing. In males previously given extended-release buprenorphine, the response to increasing doses of standard buprenorphine is altered. Data presented as means of 12 rats (6 males, 6 females) +/-S.E.M and analyzed by Welch’s unpaired t-tests followed by Bonferroni post hoc correction. *P < 0.05, **P < 0.01. A) Efficacy in response to standard buprenorphine at 1 mg/kg and 3 mg/kg was significantly decreased in rats previously exposed to extended-release buprenorphine (Bupe ER) vs. those exposed to saline. B) In female subjects analyzed alone, there is no significant difference at any dose of standard buprenorphine. C) In male subjects analyzed alone, the analgesic efficacy of 1 and 3 mg/kg buprenorphine is significantly reduced in rats previously exposed to extended-release buprenorphine vs. those exposed to saline.

To evaluate if a single administration of extended-release buprenorphine induced acute opioid tolerance, we examined the tail flick latency as it changed upon administration of increasing doses of standard buprenorphine (the dose-response curve). We tested the subjects which had received saline or extended-release buprenorphine (Fig 6) 48 hours after this administration event (Fig 7).

Compared to rats previously exposed only to saline, rats that previously received extended-release buprenorphine (1 mg/kg SC) displayed significantly reduced analgesic efficacy of standard buprenorphine (buprenorphine in its aqueous formulation) when it was given at 1 mg/kg and at 3 mg/kg (Fig 7A, both P < 0.01). There was no observed difference in efficacy at the lower doses of buprenorphine.

In female subjects analyzed alone (Fig 7B), there was no significant difference at any time point between rats previously administered extended-release buprenorphine versus subjects administered saline control (all P > 0.17).

In male subjects analyzed alone (Fig 7C), there is a significant decrease in analgesic efficacy at the 1 mg/kg dose of standard buprenorphine (P < 0.05) and the 3 mg/kg dose of standard buprenorphine (P < 0.01); there was less antinociceptive effect in male subjects previously administered extended-release buprenorphine. There was no observed difference in efficacy at the lower doses of buprenorphine (all P > 0.99).

These data support that, compared to rats which previously received only saline, rats previously administered extended-release buprenorphine at the recommended clinical dose of 1 mg/kg s.c. display an altered analgesic response to subsequent administration of standard buprenorphine. When data were examined by sex, this effect was observed here in males only, suggesting that female subjects may be less likely than male subjects to develop opioid analgesic tolerance to 1 mg/kg extended-release buprenorphine. Alternatively, in females, the acute opioid tolerance may not have persisted to 48 hours after initial exposure, or the dosing of standard buprenorphine used may not have reached a sufficiently high level to override tolerance to produce a measurable anti-nociceptive event.

Taken together, these thermal nociceptive data demonstrate that acute opioid tolerance is induced in rats after a single administration of extended-release buprenorphine at the clinically recommended dose (1 mg/kg SC).

## 4. Discussion

This study was conducted to evaluate duration of anti-hypersensitivity of extended-release buprenorphine in the rat. These data demonstrate short-lived analgesia in rats after extended-release buprenorphine has been administered in an inflammatory pain state, returning to baseline hypersensitivity by 24 hours after administration in sex-segregated data. When compared to a saline control, the female subjects demonstrated anti-hyperalgesia whereas male subjects did not in sex-segregated data, again in an inflammatory pain state. In rats with an incisional injury, extended-release buprenorphine had no effect on mechanical thresholds at any time point in both male and female subjects. Additionally, across both models of pain, mechanical withdrawal thresholds did not decrease for the contralateral paws, indicating a lack of general, centrally mediated opioid-induced hypersensitivity, or increase for the contralateral paw, indicating a lack of an overall sedative effect of the partial opioid agonist buprenorphine at this formulation and dose. When rats were repeatedly exposed to buprenorphine (first given extended-release buprenorphine and then challenged with standard buprenorphine), we observed decreased efficacy of standard buprenorphine as assessed by the dose-response curve. Sex-segregated data indicated this tolerance may be specific to male subjects. Return of ipsilateral tactile hypersensitivity, lack of centrally mediated hypersensitivity, and decreased response to buprenorphine indicate acute development of opioid tolerance in the rat after a single exposure, consistent with our observations in the mouse [16].

Per the Animal Medicinal Drug Use Clarification Act of 1994 and the associated regulations (21 Code of Federal Regulations, Part 530), extralabel use of drugs (i.e. administering them at a dose or frequency other than that listed on the label) can only be lawfully authorized by a veterinarian who has a veterinary-client-patient relationship (VCPR) with the owner and the animal. Recommended doses for extended-release buprenorphine range from 1–1.2 mg/kg [22–24]. Since this drug is labeled for use in rats at a specific dose and frequency, lay people administering this drug are legally obligated to follow those instructions exactly unless a veterinarian examining the animal prescribes some other dose. Usage of the label dose at the label frequency is anticipated by manufacturers and regulators to be the norm rather than the exception. For these reasons we selected the label dose of 1 mg/kg extended-release buprenorphine delivered subcutaneously as our dose and route of administration for testing in this study.

Extended-release buprenorphine administered at 1 mg/kg provides blood levels of buprenorphine in rats in excess of 1 ng/mL for 72 hours[25]. In thermal assays such as radiant heat tail flick latency[26] and infrared plantar test, buprenorphine levels over 1 ng/mL in blood are associated with antinociception in rats[27]. The therapeutic blood level of buprenorphine in rats remains undetermined for inflammatory and post-operative pain states, both of which are more clinically relevant to the types of pain that may be experienced by research subjects than thermal nociception. From the data obtained in these studies, buprenorphine persistence in the blood may not be correlated with duration of analgesic effect in post-operative pain states and inflammatory pain states.

The observation of analgesic tolerance developing after exposure to extended-release buprenorphine is consistent with the very earliest report [28] on buprenorphine hydrochloride, documenting that mice previously given buprenorphine for seven days developed tolerance to subsequent administration of buprenorphine and cross-tolerance to subsequent delivery of morphine. In a work using the plantar incision model in male rats, animals were given a single administration of buprenorphine and approximately a week later the tail flick responses after morphine administration were assessed; in this work only a slight reduction in tail flick latency was observed, but repeated exposure to buprenorphine was not assessed [3]. Rats have also been shown to develop tolerance and cross-tolerance to buprenorphine following seven days of opioid exposure (morphine hydrochloride 10 mg/kg or buprenorphine hydrochloride 0.25 mg/kg) on anxiolytic assessments, which is germane due to the fact that specific brain regions (the periaqueductal gray and the amygdala) are involved in both nociceptive and fear processing [29]. Data obtained from assessing buprenorphine hydrochloride in an aqueous formulation cannot be directly compared to studies assessing extended-release buprenorphine, including our work as described here.

Opioid tolerance in response to chronic opioid exposure has been discussed by researchers since the 1950s [30]. It has also been established that a single injection of opioid can trigger reduction of antinociceptive efficacy (opioid tolerance) within hours, which has been described as acute opioid tolerance [16,31–34]. An evaluation of a microparticulate formulation intended to provide a poly-lactic-co-glycolic acid-based depot of buprenorphine given at 1.2 mg/kg SC in mice produced an “initial burst release” of up to 30% of the buprenorphine, with complete release by seven days [35]. It is highly likely that a similar initial burst release occurs with this formulation of extended-release buprenorphine, triggering the observed tolerance.

The assertion that 1 ng/mL of buprenorphine in blood represents a minimum therapeutic level for rats is based on patient reports of pain relief at 1 ng/mL of plasma buprenorphine and on pharmacokinetic-pharmacodynamic modeling using thermal assays in rats [27]. Buprenorphine hydrochloride was shown to persist in the blood for up to 200 minutes after intravenous administration of 0.1 mg/kg buprenorphine in male rats, and latency to tail flick reached the 10 second cut-off time as long as 100–150 minutes after administration [26]. Pharmacokinetic analysis of various buprenorphine prodrugs in lipid formulations demonstrated measurable blood levels (above 1 ng/mL) for at least 72 hours with analgesic efficacy in male rats for up to 70 hours after injection, as determined by a plantar heat assay [36]. Our data similarly indicate that extended-release buprenorphine provides antinociceptive effect for rats experiencing acute thermal nociception. However, our data obtained in the inflammatory and post-operative pain states indicate that it is not feasible to generalize findings from one nociceptive model to another in accordance with blood levels.

Data examining sex differences and development of tolerance to buprenorphine in post-operative assays or inflammatory assays is extremely limited; most of the tolerance literature relies on thermal nociceptive assays and behavior. In one study [37] using the CFA model, male rats were administered 0.009 mg/kg buprenorphine SC twice daily for 4 days, then given buprenorphine 0.003 mg/kg intravenously and tested for tolerance of pressure using a Randall-Selitto device to deliver quantified pressure to the hindpaw; while tolerance was observed, these data were not compared to data from females. Sex differences in analgesic response to morphine on a 49°C warm-water tail flick assay have been documented in Sprague Dawley rats, with males being 2.4-fold more sensitive to its effects than females [38]. In Lewis rats and in F344 rats, the ED_50_ (the dose or amount of drug that produces a therapeutic response or desired effect in 50% of the subjects) values for buprenorphine hydrochloride when tested for tail flick latency in a 52°C water bath were significantly lower for males than for females, with buprenorphine 4–12 times more potent in males of these strains [39]. This sex difference occurs across a range of water bath temperatures (50–55°C) in nine strains of rats [40]. This supports the idea that our dose-response testing of buprenorphine in Sprague-Dawley female rats may not have escalated to a sufficiently high dose to produce differences in antinociception between those previously given extended-release buprenorphine versus those previously given saline.

To our knowledge this is the first assessment in the rat demonstrating development of opioid tolerance following one administration of extended-release buprenorphine.

## 5. Conclusions

We found that 1 mg/kg SC extended-release buprenorphine provided an anti-hyperalgesic effect for a short duration in an inflammatory model of pain but was completely ineffective in an incisional model of post-surgical pain; this dose induced opioid tolerance as determined by standard buprenorphine delivered two days later. Regarding the speed of development of acute opioid tolerance, the persistence of opioid tolerance, or the level of opioid exposure necessary to overcome tolerance, there may be a sex difference in rats which may additionally be influenced by type of nociception. Further exploration is needed.

Together, these studies demonstrate that one administration of extended-release buprenorphine at 1 mg/kg, a clinically recommended therapeutic level, confers a short duration of analgesic efficacy in the rat, likely influenced by a rapid initiation of opioid tolerance. Additional doses of buprenorphine would not be likely to provide equivalent analgesia. Development of an extended-release analgesic, especially of buprenorphine, able to confer long-term analgesia without the induction of opioid tolerance is warranted.

## Abbreviations

ANOVA: analysis of variance
SEM: standard error of the mean
MPE: maximal percent efficacy
TL: tail latency
s: seconds
ng: nanograms
mg: milligrams
g: grams
kg: kilograms
mL: milliliters
C: Celsius
SC: subcutaneous
CFA: complete Freund’s adjuvant
ER: extended-release
SR: sustained-release
ER Bupe: extended-release buprenorphine
NSAIDs: nonsteroidal anti-inflammatory drugs
